# Supplementary material for: AN INTERPRETABLE GENERATIVE MULTIMODAL NEUROIMAGING-GENOMICS FRAMEWORK FOR DECODING ALZHEIMER’S DISEASE
Source: ArXiv. 2025 Feb 4:arXiv:2406.13292v3. Originally published 2024 Jun 19. Preprint. [Version 3] (PMC11213156)
Supplement: Supplement 1 [file NIHPP2406.13292V3-supplement-1.pdf]

arXiv:2406.13292v3 [q-bio.QM] 4 Feb 2025

Supplementary Materials: An interpretable generative multimodal neuroimaging-genomics framework for decoding Alzheimer's disease

Giorgio Dolci[1,2,3], Federica Cruciani[2], Md Abdur Rahaman[3], Anees Abrol[3], Jiayu Chen[3], Zening Fu[3],
Ilaria Boscolo Galazzo[2], Gloria Menegaz[2,+], and Vince D. Calhoun[3,+],
for the Alzheimer's Disease Neuroimaging Initiative*

[1]Department of Computer Science, University of Verona, Verona, Italy
[2]Department of Engineering for Innovation Medicine, University of Verona, Verona, Italy
[3]Tri-Institutional Center for Translational Research in Neuroimaging and Data Science (TReNDS), Georgia State University, Georgia Institute of Technology, Emory University, Atlanta, GA, USA

[+]V.D. Calhoun and G. Menegaz equally contributed as last authors to this work.
[*]Data used in preparation of this article were obtained from the Alzheimer's Disease Neuroimaging Initiative (ADNI) database (adni.loni.usc.edu). As such, the investigators within the ADNI contributed to the design and implementation of ADNI and/or provided data but did not participate in analysis or writing of this report. A complete listing of ADNI investigators can be found at:
`http://adni.loni.usc.edu/wp-content/uploads/how_to_apply/ADNI_Acknowledgement_List.pdf`

February 5, 2025

Materials and Methods

Preprocessing quality control of MRI

A thorough quality control (QC) was performed to retain scans with good normalization to the standard MNI space, which involved discarding sMRI and fMRI images that exhibited low correlation with individual and/or group-level masks. In this spatial correlation process, we first calculated subject-level masks using the subject MRI scans (only the first volume in case of fMRI) by setting the brain voxels to 1 if the values of these voxels were greater than 80% of the average value across whole-brain voxels, and 0 otherwise. Next, after computing the subject-level masks, we calculated a group mask by setting the voxels to 1 for which at least 70% of the subject-level masks had a value of 1. Lastly, we examined the spatial correlations of the subject and group level masks and retained subjects that showed a correlation value greater than 0.85. Additionally, for fMRI, scans with larger head motion parameters ($> 3°$ rotations and > 3 mm translations) were discarded.

Training scheme additional information

The weights of the CNNs and classifier, used in both bi-modal models and the final framework, were initialed as follows: the 3D CNN for sMRI, the 1D CNN for SNPs, and the classifier using the Xavier Uniform distribution, while Xavier Normal distribution was used for the 1D CNN for rs-fMRI. The weights of the generators and discriminators were initialized following a Xavier Normal distribution for both the sMRI-fMRI-cGAN and sMRI-SNPs-cGAN. The weights initialization was independent between Step 1 and Step 2.

No data leakage was present between the training Step 1 (bi-modal models and cGANs) and the training Step 2 (full multimodal framework) since Step 2 involved end-to-end training using subjects with no missing modalities (i.e., using input data for these subjects only) and generative models trained in Step 1b for deriving latent features for subjects with missing modalities only. The full cohort of subjects was not used to train the bi-modal models and corresponding

cGANs (Step 1a and 1b), only relying on those subjects sharing the modalities under analysis (sMRI and rs-fMRI for sMRI-fMRI-NN/cGAN, and sMRI and SNPs for sMRI-SNPs-NN/cGAN).

Results

Classification comparison with baseline approaches

Table 1 summarizes the performance comparison of the proposed framework with the baseline methods. Regarding Task 1, AD detection, our proposed framework showed higher performance with respect to the baseline methods used for comparison. Additionally, the ensemble SVM model with mean imputation (SVM μ) and SVM with zero imputation (SVM 0) showed higher performance in Task 1 than random forest (considering both imputation methods). On the other hand, random forest with mean imputation (RF μ) and zero imputation (RF 0) strategies achieved higher performance values in Task 2 with respect to SVM approaches. Additionally, in Task 2, the proposed model outperformed in terms of accuracy the baseline methods, but both random forest approaches achieved a precision value higher than the proposed framework.

Table 1: Performance comparison of the proposed framework with two baseline methods, SVM and random forest (RF), and two imputation strategies, mean imputation (μ) and zero imputation (0).

	Task 1			Task 2		
	Accuracy	Precision	Recall	Accuracy	Precision	Recall
SVM μ	0.699 ± 0.01	0.852 ± 0.09	0.149 ± 0.05	0.690 ± 0.01	0.399 ± 0.16	0.074 ± 0.06
SVM 0	0.689 ± 0.02	0.900 ± 0.08	0.105 ± 0.06	0.688 ± 0.01	0.325 ± 0.15	0.041 ± 0.06
RF μ	0.663 ± 0.01	0.533 ± 0.45	0.018 ± 0.02	0.698 ± 0.01	0.804 ± 0.17	0.028 ± 0.01
RF 0	0.663 ± 0.01	0.800 ± 0.40	0.015 ± 0.01	0.694 ± 0.01	0.836 ± 0.14	0.015 ± 0.01
Our	0.926 ± 0.02	0.910 ± 0.05	0.876 ± 0.03	0.711 ± 0.01	0.558 ± 0.03	0.610 ± 0.03

Table 2: In this Table are reported the correspondences acronym - full name of the sMRI brain regions under analysis.

Acronym	Full name	Acronym	Full name	Acronym	Full name
Ins	Insular Cortex	OFG	Occipital Fusiform Gyrus	TP	Temporal Pole
COpC	Central Opercular Cortex	ScC	Subcallosal Cortex	POpC	Parietal Operculum Cortex
CGp	Cingulate Gyrus, posterior division	Thl	Thalamus	PcC	Precuneous Cortex
Cau	Caudate	FOC	Frontal Orbital Cortex	Put	Putamen
PhGa	Parahippocampal Gyrus, anterior division	Pall	Pallidum	PaGp	Parahippocampal Gyrus, posterior division
Hipp	Hippocampus	LG	Lingual Gyrus	Amy	Amygdala
TFCp	Temporal Fusiform Cortex, posterior division	Acc	Accumbens	TOF	Temporal Occipital Fusiform Cortex

Group-based statistical IG analysis

Neuroimaging modalities

Supplementary Figure 1A shows the statistical analysis results of sMRI data. The heatmap reports the Bonferroni corrected p-values (6 pairwise comparisons) in logarithmic scale (yellow/red correspond to higher/lower p-value, and white means non-significant). While supplementary Figure 1B shows the statistical analysis results of rs-fMRI data.

Table 3: In this Table are reported the 53 ICs present in the sFNC matrices with corresponding brain region name, network at which it belongs to, and spatial location in the brain along X, Y, and Z axis. SC=Sub-cortical; AU=Auditory; SM=sensorimotor; VI=visual; CC=cognitive-control; DM=default-mode; and CB=cerebellar. In *italic* are highlighted the brain regions present in the fMRI connectograms of Results section.

IC ID	Brain region	Network	X	Y	Z	IC ID	Brain region	Network	X	Y	Z
1	Caudate	SC	6.5	10.5	5.5	26	Inferior parietal lobule	CC	45.5	-61.5	43.5
2	Subthalamus/hypothalamys	SC	-2.5	-13.5	-1.5	27	Insula	CC	-30.5	22.5	-3.5
3	Putamen	SC	-26.5	1.5	-0.5	28	*Superior medial frontal gyrus*	CC	-0.5	50.5	29.5
4	Caudate	SC	21.5	10.5	-3.5	29	Inferior frontal gyrus	CC	-48.5	34.5	-0.5
5	Thalamus	SC	-12.5	-18.5	11.5	30	Right inferior frontal gyrus	CC	53.5	22.5	13.5
6	Superior temporal gyrus	AU	62.5	-22.5	7.5	31	Middle frontal gyrus	CC	-41.5	19.5	26.5
7	Middle temporal gyrus	AU	-42.5	-6.5	10.5	32	Inferior parietal lobule	CC	-53.5	-49.5	43.5
8	*Postcentral gyrus*	SM	56.5	-4.5	28.5	33	*Left inferior parietal lobule*	CC	44.5	-34.5	46.5
9	*Left postcentral gyrus*	SM	-38.5	-22.5	56.5	34	Supplementary motor area	CC	-6.5	13.5	64.5
10	*Paracentral lobule*	SM	0.5	-22.5	65.5	35	Superior frontal gyrus	CC	-24.5	26.5	49.5
11	*Right postcentral gyrus*	SM	38.5	-19.5	55.5	36	Middle frontal gyrus	CC	30.5	41.5	28.5
12	*Superior parietal lobule*	SM	-18.5	-43.5	65.5	37	Hippocampus	CC	23.5	-9.5	-16.5
13	*Paracentral lobule*	SM	-18.5	-9.5	56.5	38	*Left inferior parietal lobule*	CC	45.5	-61.5	43.5
14	*Precentral gyrus*	SM	-42.5	-7.5	46.5	39	Middle cingulate cortex	CC	-15.5	20.5	37.5
15	*Superior parietal lobule*	SM	20.5	-63.5	58.5	40	Inferior frontal gyrus	CC	39.5	44.5	-0.5
16	*Postcentral gyrus*	SM	-47.5	-27.5	43.5	41	Middle frontal gyrus	CC	-26.5	47.5	5.5
17	*Calcarine gyrus*	VI	-12.5	-66.5	8.5	42	*Hippocampus*	CC	-24.5	-36.5	1.5
18	*Middle occipital gyrus*	VI	-23.5	-93.5	-0.5	43	Precuneus	DM	-8.5	-66.5	35.5
19	Middle temporal gyrus	VI	48.5	-60.5	10.5	44	*Precuneus*	DM	-12.5	-54.5	14.5
20	*Cuneus*	VI	15.5	-91.5	22.5	45	*Anterior cingulate cortex*	DM	-2.5	35.5	2.5
21	*Right middle occipital gyrus*	VI	38.5	-73.5	6.5	46	Posterior cingulate cortex	DM	-5.5	-28.5	26.5
22	*Fusiform gyrus*	VI	29.5	-42.5	-12.5	47	*Anterior cingulate cortex*	DM	-9.5	46.5	-10.5
23	*Inferior occipital gyrus*	VI	-36.5	-76.5	-4,5	48	*Precuneus*	DM	-0.5	-48.5	49.5
24	*Lingual gyrus*	VI	-8.5	-81.5	-4.5	49	Posterior cingulate cortex	DM	-2.5	54.5	31.5
25	*Middle temporal gyrus*	VI	-44.5	-57.5	-7.5	50	*Cerebellum*	CB	-30.5	-54.5	-42.5
						51	*Cerebellum*	CB	-32.5	-79.5	-37.5
						52	Cerebellum	CB	20.5	-48.5	-40.5
						53	*Cerebellum*	CB	30.5	-63.5	-40.5

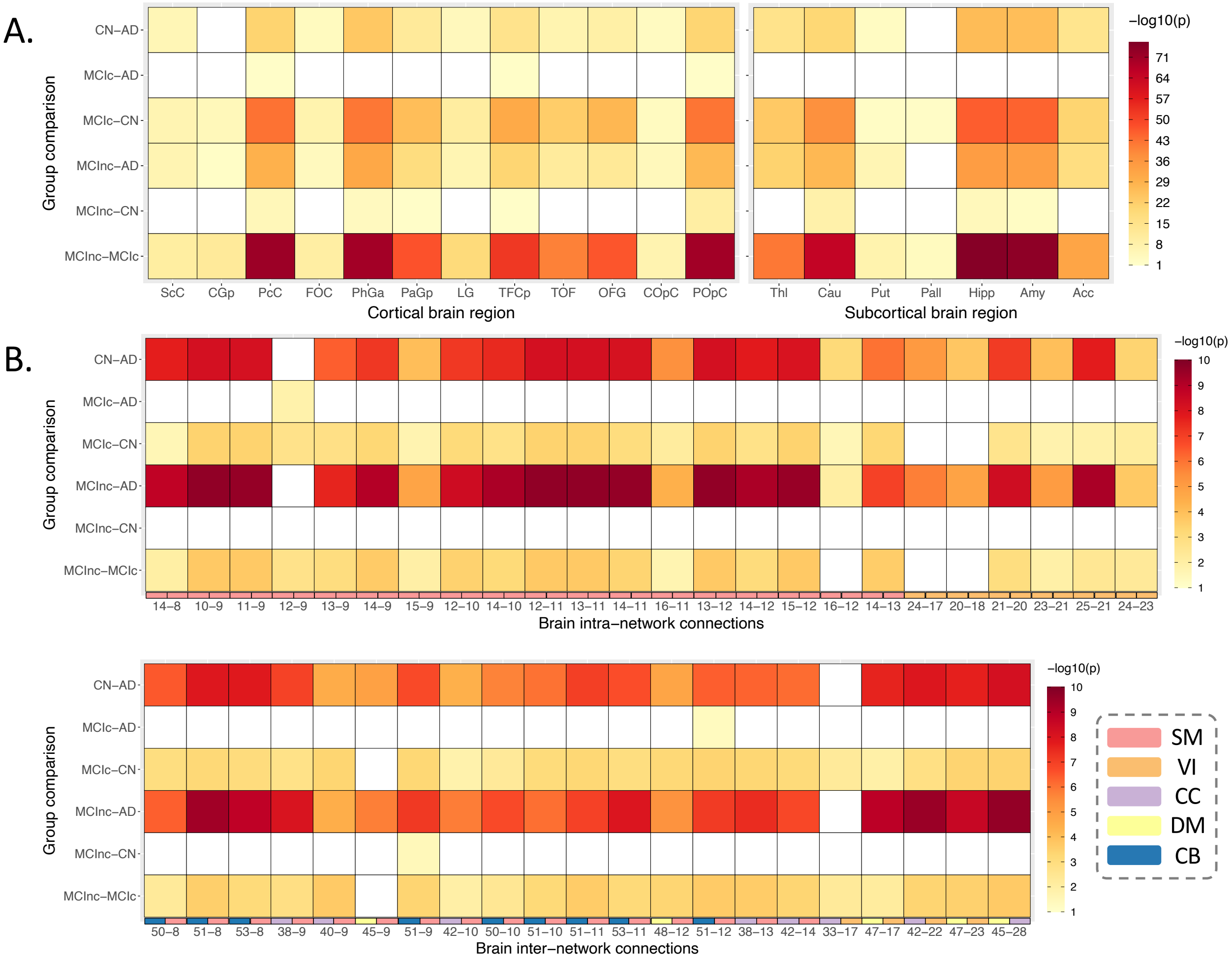

Figure 1: Overview of the neuroimaging statistical analysis. A. sMRI-IG Wilcoxon test results for each group comparison and each brain region, divided into cortical and subcortical regions; B. fMRI-IG Wilcoxon test results for each group comparison for each relevant brain connection divided into extra- (upper) and intra-network (lower) connectivity. The *p*-values for B. and C. are reported in negative logarithmic scale, dark red the most significant. White cells represent no statistical significance.

Table 4: Most significant biological processes for AD patients obtained from the analysis of the most relevant SNPs with positive IG attributions. In this Table are shown the biological processes, their raw p-value, and the overlap genes. * indicates statistically significant biological processes after Bonferroni correction.

Biological processes	GO term	p-value	Overlap genes
Intracellular transport*	GO:0046907	0.000044 ($p_{bonf} = 0.0225$)	BLOC1S3; KLC3; TOMM40; PICALM; MME; NUP88; TMEM106B; NSF; NUP43; BIN1; RAB12; APOE; SORL1; CLU
Regulation of protein-containing complex assembly*	GO:0043254	0.000080 ($p_{bonf} = 0.0413$)	PTK2B; MARK4; FNIP2; PLCG2; BIN1; APOE; SORL1; TREM2; CLU
Establishment of localization in cell*	GO:0051649	0.000086 ($p_{bonf} = 0.0446$)	BLOC1S3; KLC3; TOMM40; PICALM; MME; NUP88; TMEM106B; NSF; NUP43; BIN1; RAB12; APOE; SORL1; NECTIN2; CLU
Cell development*	GO:0048468	0.000091 ($p_{bonf} = 0.0470$)	BLOC1S3; KLC3; PICALM; PTK2B; ALDH1A2; TNXB; TMEM106B; FCER1G; OOSP2; PLCG2; IGSF23; BIN1; ERCC2; RELB; APOE; ETV1; NECTIN2; TREM2; CLU
Cell projection organization	GO:0030030	0.000253	BLOC1S3; KLC3; PICALM; PTK2B; TNXB; TMEM106B; MARK4; CDH13; APOE; ETV1; MTSS2; NECTIN2; TREM2
Plasma membrane bounded cell projection organization	GO:0120036	0.000253	BLOC1S3; KLC3; PICALM; PTK2B; TNXB; TMEM106B; MARK4; CDH13; APOE; ETV1; MTSS2; NECTIN2; TREM2
Membrane organization	GO:0061024	0.000253	TOMM40; PICALM; RABEP1; NSF; APOA2; BIN1; CR1; RAB12; APOE; MTSS2; NECTIN2; TREM2; CLU
Developmental maturation	GO:0021700	0.000417	BLOC1S3; PICALM; PTK2B; ALDH1A2; SLC24A4; OOSP2; ERCC2
Positive regulation of endocytosis	GO:0045807	0.000417	FCER1G; PLCG2; APOA2; BIN1; APOE; TREM2; CLU
Regulation of vesicle-mediated transport	GO:0060627	0.000427	PICALM; NSF; FCER1G; PLCG2; APOA2; BIN1; RAB12; CDH13; APOE; SORL1; TREM2; CLU
Endomembrane system organization	GO:0010256	0.000500	BLOC1S3; NSF; USP8; BIN1; CR1; USP6NL; MTSS2; NECTIN2; CLU
Nitrogen compound transport	GO:0071705	0.000741	BLOC1S3; TOMM40; RABEP1; MME; NUP88; NSF; NUP43; CR1; RAB12; APOE; SORL1; CLU
Organelle localization	GO:0051640	0.000771	BLOC1S3; PICALM; NUP88; TMEM106B; CLU; BIN1; RAB12; NECTIN2
Cellular component organization or biogenesis	GO:0071840	0.000792	BLOC1S3; KLC3; TOMM40; PICALM; RABEP1; MME; NUP88; PTK2B; ALDH1A2; PHB1; TNXB; TMEM106B; NSF; MARK4; KCTD1; CCDC6; CLU; MINDY2; LTBP4; APOA2; USP8; BIN1; CR1; ERCC2; USP6NL; RAB12; GEMIN7; CDH13; APOE; ETV1; MTSS2; NECTIN2; TREM2; CLU
Localization	GO:0051179	0.000950	BLOC1S3; KLC3; TOMM40; PICALM; RABEP1; MME; NUP88; TMEM106B; NSF; FCER1G; SLC24A4; CLU; PLCG2; LTBP4; APOA2; NUP43; BIN1; CR1; USP6NL; RAB12; NBEAL1; CDH13; APOE; SORL1; NECTIN2; TREM2; CLU

Table 5: Most significant biological processes for MCIc patients obtained from the analysis of the most relevant SNPs with positive IG attributions. In this Table are shown the biological processes, their raw p-value, and the overlap genes.

Biological processes	GO term	p-value	Overlap genes
Positive regulation of amide metabolic process	GO:0034250	0.00109	PICALM; RPS27L; PTK2B; APOE; CLU
Protein-lipid complex assembly	GO:0065005	0.00109	APOC1; ABCA7; APOA2; BIN1; APOE
Regulation of amide metabolic process	GO:0034248	0.00128	PICALM; RPS27L; PTK2B; ABCA7; BIN1; APOE; CELF1; CLU
Positive regulation of endocytosis	GO:0045807	0.00147	FCER1G; ABCA7; PLCG2; APOA2; BIN1; APOE; CLU
Plasma lipoprotein particle assembly	GO:0034377	0.00436	APOC1; ABCA7; APOA2; APOE
Regulation of endocytosis	GO:0030100	0.00453	PICALM; APOC1; FCER1G; ABCA7; PLCG2; APOA2; BIN1; APOE; CLU
Sterol transport	GO:0015918	0.00519	APOC1; ABCA7; APOA2; APOE; CLU
Organic hydroxy compound transport	GO:0015850	0.00519	APOC1; ABCA7; APOA2; APOE; CLU
Cholesterol transport	GO:0030301	0.00519	APOC1; ABCA7; APOA2; APOE; CLU
Protein-lipid complex organization	GO:0071825	0.00519	APOC1; ABCA7; APOA2; BIN1; APOE
Regulation of lipid metabolic process	GO:0019216	0.00841	LACTB; APOC1; ABCA7; PLCG2; APOA2; EPHX2; APOE
Negative regulation of amide metabolic process	GO:0034249	0.01440	ABCA7; BIN1; APOE; CELF1; CLU
Negative regulation of amyloid precursor protein catabolic process	GO:1902992	0.01440	PICALM; ABCA7; BIN1; APOE; CLU
Monoatomic cation homeostasis	GO:0055080	0.01440	PICALM; TMEM106B; SLC24A4; TSPOAP1; APOE
Intracellular monoatomic cation homeostasis	GO:0030003	0.01440	PICALM; TMEM106B; SLC24A4; TSPOAP1; APOE

Table 6: Most significant biological processes for MCInc patients obtained from the analysis of the most relevant SNPs with positive IG attributions. In this Table are shown the biological processes, their raw *p*-value, and the overlap genes.

Biological processes	GO term	p-value	Overlap genes
T cell activation	GO:0042110	0.00695	FCER1G; TREML2; SPI1; RELB
Lymphocyte activation involved in immune response	GO:0002285	0.00695	FCER1G; PLCG2; ERCC1; RELB
Negative regulation of hydrolase activity	GO:0051346	0.00695	PICALM; APOA2; BIN1; CR1
T cell differentiation	GO:0030217	0.00836	FCER1G; SPI1; RELB
Negative regulation of peptidase activity	GO:0010466	0.00836	PICALM; BIN1; CR1
Negative regulation of endopeptidase activity	GO:0010951	0.00836	PICALM; BIN1; CR1
Adaptive immune response based on somatic recombination of immune receptors built from immunoglobulin superfamily domains	GO:0002460	0.01170	FCER1G; CR1L; HLA-DRB1; ERCC1; CR1; RELB
Immune effector process	GO:0002252	0.01800	FCER1G; CR1L; PLCG2; ACE; ERCC1; CR1; RELB
Leukocyte mediated immunity	GO:0002443	0.02020	FCER1G; CR1L; ACE; ERCC1; CR1
Male gonad development	GO:0008584	0.02850	ACE; ERCC1; PLEKHA1
Male sex differentiation	GO:0046661	0.02850	ACE; ERCC1; PLEKHA1
Development of primary male sexual characteristics	GO:0046546	0.02850	ACE; ERCC1; PLEKHA1
Antigen processing and presentation of peptide antigen	GO:0048002	0.02850	FCER1G; ACE; HLA-DRB1
Lymphocyte activation	GO:0046649	0.03080	FCER1G; TREML2; PLCG2; ERCC1; SPI1; RELB
Positive regulation of lymphocyte activation	GO:0051251	0.03400	BLOC1S3; HLA-DRB1; SMARCD3; CR1; SPI1

Table 7: Performance comparison of our model with other state-of-the-art methods dealing with missing modalities for AD detection and MCI conversion tasks.

(a) AD detection task. Accuracy (ACC), precision (PRE), and recall (REC) metrics are reported on the test set or averaged during the cross-validation phase ($mean \pm std$).

Authors	Modalities	Study cohort	Input data	Missing data rate	Missing data management	ACC	REC	PRE	XAI
Venugopalan et al. [1]	sMRI, Genetics, Clinics	1406 PAT** 598 CN	Full MRI volume WGS data, Set of clinical scores	~ 67% missing sMRI ~ 61% missing SNP	Zero filling	0.630* 0.780†	0.570* 0.780†	0.620* 0.770†	Yes
Liu et al. [2]	sMRI, FDG-PET	85 AD 90 CN	ROI based features	50% missing PET	Auto-Encoder for complementing complete and incomplete latent representation	0.836 ± 0.08	n.d.	n.d.	No
Gao et al. [3]	sMRI, FDG-PET	352 AD 427 CN	sMRI and PET volumes	30% missing PET	Task-induced pyramid and attention GAN	0.927	0.917	n.d.	No
Gao et al. [4]	sMRI, FDG-PET, T2-MRI	352 AD 427 CN	sMRI, PET, T2-MRI volumes	n.d. % missing data	Multi-level guided GAN	0.944	0.930	n.d.	Yes
Zhang et al. [5]	sMRI, FDG-PET	345 AD 531 CN	sMRI and PET volumes	Generated synthetic PET	GAN-based multiple convolution U-Net	0.981	n.d.	n.d.	No
Ye et al. [6]	sMRI, FDG-PET	160 AD 210 CN	ROI based features for VBM, FDG	50% missing PET	GAN with attention layer in latent space	0.914 ± 0.19	0.881 ± 0.11	n.d.	Yes
Tu et al. [7]	sMRI, FDG-PET	255 AD 124 CN	sMRI and PET volumes	Generated synthetic PET	Consistent manifold projection GAN	0.980 ± 0.003	0.932 ± 0.012	n.d.	No
Zhang et al. [8]	sMRI, FDG-PET	n.d. AD n.d. CN	sMRI and PET volumes	Generated synthetic PET	GAN pyramidal attention mechanism	0.934	0.975	n.d.	Yes
Our	sMRI, fMRI, Genetics	332 AD 664 CN	GM volume sFNC matrix, SNPs	~ 59% missing fMRI ~ 57% missing SNPs	Multiple cGANs to generate missing latent representation	0.926 ± 0.02	0.876 ± 0.03	0.910 ± 0.05	Yes

(b) MCI conversion prediction. Accuracy (ACC), precision (PRE), and recall (REC) metrics are reported on the test set.

Authors	Modalities	Study cohort	Input data	Missing data rate	Missing data management	Trained on the specific task	ACC	REC	PRE	XAI
Ritter et al. [9]	10 modalities (imaging and clinics)	86 MCIc 237 MCInc	Tabular features	~ 8% missing features	Mean imputation and imputation by the EM	Yes	0.670	n.d.	n.d.	No
Cai et al. [10]	sMRI, FDG-PET	76 MCIc 128 MCInc	GM volume PET volumes	50% missing PET	3D encoder-decoder network to generate the full PET volume	Yes	0.657	n.d.	n.d.	No
Zhou et al. [11]	sMRI, FDG-PET Genetics	157 MCIc 205 MCInc	ROI based features for PET and MRI, SNPs	51% missing PET	Latent representation learning without missing data imputation	Yes	0.743	n.d.	n.d.	Yes
Gao et al. [3]	sMRI, FDG-PET	234 MCIc 342 MCInc	sMRI and PET volumes	30% missing PET	Task-induced pyramid and attention GAN	Yes	0.753	0.708	n.d.	No
Gao et al. [4]	sMRI, FDG-PET, T2-MRI	234 MCIc 342 MCInc	sMRI, PET, T2-MRI volumes	n.d. % missing data	Multi-level guided GAN	Yes, pre-training on CN vs AD	0.778	0.754	n.d.	No
Tu et al. [7]	sMRI, FDG-PET	121 MCIc 321 MCInc	sMRI and PET volumes	Generated synthetic PET	Consistent manifold projection GAN	Yes	0.923 ± 0.010	0.957 ± 0.014	n.d.	No
Our	sMRI, fMRI, Genetics	289 MCIc 646 MCInc	GM volume sFNC matrix, SNPs	~ 76% missing fMRI ~ 45% missing SNPs	Multiple cGANs to generate missing latent representation	No	0.711 ± 0.01	0.610 ± 0.03	0.558 ± 0.03	Yes

**PAT = 266 AD and 699 MCI, * CN/PAT (AD+MCI) classification relying on sMRI and SNPs, † Three classes classification, CN/MCI/AD relying on the three modalities, n.d. = not declared

References

[1] Janani Venugopalan, Li Tong, Hamid Reza Hassanzadeh, and May D Wang. Multimodal deep learning models for early detection of alzheimer's disease stage. *Scientific reports*, 11(1):3254, 2021.

[2] Yanbei Liu, Lianxi Fan, Changqing Zhang, Tao Zhou, Zhitao Xiao, Lei Geng, and Dinggang Shen. Incomplete multi-modal representation learning for alzheimer's disease diagnosis. *Medical Image Analysis*, 69:101953, 2021.

[3] Xingyu Gao, Feng Shi, Dinggang Shen, and Manhua Liu. Task-induced pyramid and attention gan for multimodal brain image imputation and classification in alzheimer's disease. *IEEE journal of biomedical and health informatics*, 26(1):36–43, 2021.

[4] Xingyu Gao, Feng Shi, Dinggang Shen, and Manhua Liu. Multimodal transformer network for incomplete image generation and diagnosis of alzheimer's disease. *Computerized Medical Imaging and Graphics*, 110:102303, 2023.

[5] Jin Zhang, Xiaohai He, Linbo Qing, Feng Gao, and Bin Wang. Bpgan: Brain pet synthesis from mri using generative adversarial network for multi-modal alzheimer's disease diagnosis. *Computer Methods and Programs in Biomedicine*, 217:106676, 2022.

[6] Haizhou Ye, Qi Zhu, Yuan Yao, Yichao Jin, and Daoqiang Zhang. Pairwise feature-based generative adversarial network for incomplete multi-modal alzheimer's disease diagnosis. *The Visual Computer*, pages 1–10, 2022.

[7] Yue Tu, Shukuan Lin, Jianzhong Qiao, Yilin Zhuang, Zhiqi Wang, and Dai Wang. Multimodal fusion diagnosis of alzheimer's disease based on fdg-pet generation. *Biomedical Signal Processing and Control*, 89:105709, 2024.

[8] Mengyi Zhang, Lijing Sun, Zhaokai Kong, Wenjun Zhu, Yang Yi, and Fei Yan. Pyramid-attentive gan for multimodal brain image complementation in alzheimer's disease classification. *Biomedical Signal Processing and Control*, 89:105652, 2024.

[9] Kerstin Ritter, Julia Schumacher, Martin Weygandt, Ralph Buchert, Carsten Allefeld, John-Dylan Haynes, Alzheimer's Disease Neuroimaging Initiative, et al. Multimodal prediction of conversion to alzheimer's disease based on incomplete biomarkers. *Alzheimer's & Dementia: Diagnosis, Assessment & Disease Monitoring*, 1(2):206–215, 2015.

[10] Lei Cai, Zhengyang Wang, Hongyang Gao, Dinggang Shen, and Shuiwang Ji. Deep adversarial learning for multi-modality missing data completion. In *Proceedings of the 24th ACM SIGKDD international conference on knowledge discovery & data mining*, pages 1158–1166, 2018.

[11] Tao Zhou, Mingxia Liu, Kim-Han Thung, and Dinggang Shen. Latent representation learning for alzheimer's disease diagnosis with incomplete multi-modality neuroimaging and genetic data. *IEEE transactions on medical imaging*, 38(10):2411–2422, 2019.
