# Supplementary material for: An interpretable generative multimodal neuroimaging-genomics framework for decoding alzheimer’s disease
Source: ArXiv. 2024 Nov 14:arXiv:2406.13292v2. Originally published 2024 Jun 19. Preprint. [Version 2] (PMC11213156)
Supplement: Supplementary file 1 [file NIHPP2406.13292v2-supplement-1.pdf]

Supplementary Materials: An interpretable generative multimodal neuroimaging-genomics framework for decoding Alzheimer's disease

arXiv:2406.13292v2 [q-bio.QM] 14 Nov 2024

Giorgio Dolci[a,b,], Federica Cruciani[a*], Md Abdur Rahaman[b], Anees Abrol[b], Jiayu Chen[b], Zening Fu[b], Ilaria Boscolo Galazzo[a], Gloria Menegaz[a,**], Vince D. Calhoun[b,**], for the Alzheimer's Disease Neuroimaging Initiative[1]

[a] *Department of Engineering and Innovation Medicine, University of Verona, Verona, Italy*
[b] *Tri-Institutional Center for Translational Research in Neuroimaging and Data Science (TReNDS), Georgia State University, Georgia Institute of Technology, Emory University, Atlanta, GA, USA*

Preprocessing quality control

A thorough quality control (QC) was performed to retain scans with good normalization to the standard MNI space, which involved discarding sMRI and fMRI images that exhibited low correlation with individual and/or group-level masks. In this spatial correlation process, we first calculated subject-level masks using the subject MRI scans (only the first volume in case of fMRI) by setting the brain voxels to 1 if the values of these voxels were greater than 80% of the average value across whole-brain voxels, and 0 otherwise. Next, after computing the subject-level masks, we calculated a group mask by setting the voxels to 1 for which at least 70% of the subject-level masks had a value of 1. Lastly, we examined the spatial correlations of the subject and group level masks and retained subjects that showed a correlation value greater than 0.85. Additionally, for fMRI, scans with larger head motion parameters ($> 3°$ rotations and > 3 mm translations) were discarded.

*Corresponding author: Department of Engineering and Innovation Medicine, University of Verona, Verona, Italy.
e-mail: federica.cruciani@univr.it

**Equally contributed as last authors to this work.

[1]Data used in preparation of this article were obtained from the Alzheimer's Disease Neuroimaging Initiative (ADNI) database (adni.loni.usc.edu). As such, the investigators within the ADNI contributed to the design and implementation of ADNI and/or provided data but did not participate in analysis or writing of this report. A complete listing of ADNI investigators can be found at: `http://adni.loni.usc.edu/wp-content/uploads/how_to_apply/ADNI_Acknowledgement_List.pdf`

Results

Table 1: In this Table are reported the correspondences acronym - full name of the sMRI brain regions under analysis.

Acronym	Full name	Acronym	Full name
Ins	Insular Cortex	OFG	Occipital Fusiform Gyrus
TP	Temporal Pole	COpC	Central Opercular Cortex
ScC	Subcallosal Cortex	POpC	Parietal Operculum Cortex
CGp	Cingulate Gyrus, posterior division	Thl	Thalamus
PcC	Precuneous Cortex	Cau	Caudate
FOC	Frontal Orbital Cortex	Put	Putamen
PhGa	Parahippocampal Gyrus, anterior division	Pall	Pallidum
PaGp	Parahippocampal Gyrus, posterior division	Hipp	Hippocampus
LG	Lingual Gyrus	Amy	Amygdala
TFCp	Temporal Fusiform Cortex, posterior division	Acc	Accumbens
TOF	Temporal Occipital Fusiform Cortex		

Table 2: In this Table are reported the 53 ICs present in the sFNC matrices with corresponding brain region name, network at which it belongs to, and spatial location in the brain along X, Y, and Z axis. SC=Sub-cortical; AU=Auditory; SM=sensorimotor; VI=visual; CC=cognitive-control; DM=default-mode; and CB=cerebellar. In *italic* are highlighted the brain regions present in the fMRI connectograms of Results section.

IC ID	Brain region	Network	X	Y	Z	IC ID	Brain region	Network	X	Y	Z
1	Caudate	SC	6.5	10.5	5.5	26	Inferior parietal lobule	CC	45.5	-61.5	43.5
2	Subthalamus/hypothalamys	SC	-2.5	-13.5	-1.5	27	Insula	CC	-30.5	22.5	-3.5
3	Putamen	SC	-26.5	1.5	-0.5	28	*Superior medial frontal gyrus*	CC	-0.5	50.5	29.5
4	Caudate	SC	21.5	10.5	-3.5	29	Inferior frontal gyrus	CC	-48.5	34.5	-0.5
5	Thalamus	SC	-12.5	-18.5	11.5	30	Right inferior frontal gyrus	CC	53.5	22.5	13.5
6	Superior temporal gyrus	AU	62.5	-22.5	7.5	31	Middle frontal gyrus	CC	-41.5	19.5	26.5
7	Middle temporal gyrus	AU	-42.5	-6.5	10.5	32	Inferior parietal lobule	CC	-53.5	-49.5	43.5
8	*Postcentral gyrus*	SM	56.5	-4.5	28.5	33	*Left inferior parietal lobule*	CC	44.5	-34.5	46.5
9	*Left postcentral gyrus*	SM	-38.5	-22.5	56.5	34	Supplementary motor area	CC	-6.5	13.5	64.5
10	*Paracentral lobule*	SM	0.5	-22.5	65.5	35	Superior frontal gyrus	CC	-24.5	26.5	49.5
11	*Right postcentral gyrus*	SM	38.5	-19.5	55.5	36	Middle frontal gyrus	CC	30.5	41.5	28.5
12	*Superior parietal lobule*	SM	-18.5	-43.5	65.5	37	Hippocampus	CC	23.5	-9.5	-16.5
13	*Paracentral lobule*	SM	-18.5	-9.5	56.5	38	*Left inferior parietal lobule*	CC	45.5	-61.5	43.5
14	*Precentral gyrus*	SM	-42.5	-7.5	46.5	39	Middle cingulate cortex	CC	-15.5	20.5	37.5
15	*Superior parietal lobule*	SM	20.5	-63.5	58.5	40	Inferior frontal gyrus	CC	39.5	44.5	-0.5
16	*Postcentral gyrus*	SM	-47.5	-27.5	43.5	41	Middle frontal gyrus	CC	-26.5	47.5	5.5
17	*Calcarine gyrus*	VI	-12.5	-66.5	8.5	42	*Hippocampus*	CC	-24.5	-36.5	1.5
18	*Middle occipital gyrus*	VI	-23.5	-93.5	-0.5	43	Precuneus	DM	-8.5	-66.5	35.5
19	Middle temporal gyrus	VI	48.5	-60.5	10.5	44	*Precuneus*	DM	-12.5	-54.5	14.5
20	*Cuneus*	VI	15.5	-91.5	22.5	45	*Anterior cingulate cortex*	DM	-2.5	35.5	2.5
21	*Right middle occipital gyrus*	VI	38.5	-73.5	6.5	46	Posterior cingulate cortex	DM	-5.5	-28.5	26.5
22	*Fusiform gyrus*	VI	29.5	-42.5	-12.5	47	*Anterior cingulate cortex*	DM	-9.5	46.5	-10.5
23	*Inferior occipital gyrus*	VI	-36.5	-76.5	-4,5	48	*Precuneus*	DM	-0.5	-48.5	49.5
24	*Lingual gyrus*	VI	-8.5	-81.5	-4.5	49	Posterior cingulate cortex	DM	-2.5	54.5	31.5
25	*Middle temporal gyrus*	VI	-44.5	-57.5	-7.5	50	*Cerebellum*	CB	-30.5	-54.5	-42.5
						51	*Cerebellum*	CB	-32.5	-79.5	-37.5
						52	Cerebellum	CB	20.5	-48.5	-40.5
						53	*Cerebellum*	CB	30.5	-63.5	-40.5

Table 3: Most significant biological processes for AD patients obtained from the analysis of the most relevant SNPs with positive IG attributions. In this Table are shown the biological processes, their raw p-value, and the overlap genes. * indicates statistically significant biological processes after Bonferroni correction.

Biological processes	GO term	p-value	Overlap genes
Intracellular transport*	GO:0046907	0.000044 (p_{bonf} = 0.0225)	BLOC1S3; KLC3; TOMM40; PICALM; MME; NUP88; TMEM106B; NSF; NUP43; BIN1; RAB12; APOE; SORL1; CLU
Regulation of protein-containing complex assembly*	GO:0043254	0.000080 (p_{bonf} = 0.0413)	PTK2B; MARK4; FNIP2; PLCG2; BIN1; APOE; SORL1; TREM2; CLU
Establishment of localization in cell*	GO:0051649	0.000086 (p_{bonf} = 0.0446)	BLOC1S3; KLC3; TOMM40; PICALM; MME; NUP88; TMEM106B; NSF; NUP43; BIN1; RAB12; APOE; SORL1; NECTIN2; CLU
Cell development*	GO:0048468	0.000091 (p_{bonf} = 0.0470)	BLOC1S3; KLC3; PICALM; PTK2B; ALDH1A2; TNXB; TMEM106B; FCER1G; OOSP2; PLCG2; IGSF23; BIN1; ERCC2; RELB; APOE; ETV1; NECTIN2; TREM2; CLU
Cell projection organization	GO:0030030	0.000253	BLOC1S3; KLC3; PICALM; PTK2B; TNXB; TMEM106B; MARK4; CDH13; APOE; ETV1; MTSS2; NECTIN2; TREM2
Plasma membrane bounded cell projection organization	GO:0120036	0.000253	BLOC1S3; KLC3; PICALM; PTK2B; TNXB; TMEM106B; MARK4; CDH13; APOE; ETV1; MTSS2; NECTIN2; TREM2
Membrane organization	GO:0061024	0.000253	TOMM40; PICALM; RABEP1; NSF; APOA2; BIN1; CR1; RAB12; APOE; MTSS2; NECTIN2; TREM2; CLU
Developmental maturation	GO:0021700	0.000417	BLOC1S3; PICALM; PTK2B; ALDH1A2; SLC24A4; OOSP2; ERCC2
Positive regulation of endocytosis	GO:0045807	0.000417	FCER1G; PLCG2; APOA2; BIN1; APOE; TREM2; CLU
Regulation of vesicle-mediated transport	GO:0060627	0.000427	PICALM; NSF; FCER1G; PLCG2; APOA2; BIN1; RAB12; CDH13; APOE; SORL1; TREM2; CLU
Endomembrane system organization	GO:0010256	0.000500	BLOC1S3; NSF; USP8; BIN1; CR1; USP6NL; MTSS2; NECTIN2; CLU
Nitrogen compound transport	GO:0071705	0.000741	BLOC1S3; TOMM40; RABEP1; MME; NUP88; NSF; NUP43; CR1; RAB12; APOE; SORL1; CLU
Organelle localization	GO:0051640	0.000771	BLOC1S3; PICALM; NUP88; TMEM106B; CLU; BIN1; RAB12; NECTIN2
Cellular component organization or biogenesis	GO:0071840	0.000792	BLOC1S3; KLC3; TOMM40; PICALM; RABEP1; MME; NUP88; PTK2B; ALDH1A2; PHB1; TNXB; TMEM106B; NSF; MARK4; KCTD1; CCDC6; CLU; MINDY2; LTBP4; APOA2; USP8; BIN1; CR1; ERCC2; USP6NL; RAB12; GEMIN7; CDH13; APOE; ETV1; MTSS2; NECTIN2; TREM2; CLU
Localization	GO:0051179	0.000950	BLOC1S3; KLC3; TOMM40; PICALM; RABEP1; MME; NUP88; TMEM106B; NSF; FCER1G; SLC24A4; CLU; PLCG2; LTBP4; APOA2; NUP43; BIN1; CR1; USP6NL; RAB12; NBEAL1; CDH13; APOE; SORL1; NECTIN2; TREM2; CLU

Table 4: Most significant biological processes for MCIc patients obtained from the analysis of the most relevant SNPs with positive IG attributions. In this Table are shown the biological processes, their raw p-value, and the overlap genes.

Biological processes	GO term	p-value	Overlap genes
Positive regulation of amide metabolic process	GO:0034250	0.00109	PICALM; RPS27L; PTK2B; APOE; CLU
Protein-lipid complex assembly	GO:0065005	0.00109	APOC1; ABCA7; APOA2; BIN1; APOE
Regulation of amide metabolic process	GO:0034248	0.00128	PICALM; RPS27L; PTK2B; ABCA7; BIN1; APOE; CELF1; CLU
Positive regulation of endocytosis	GO:0045807	0.00147	FCER1G; ABCA7; PLCG2; APOA2; BIN1; APOE; CLU
Plasma lipoprotein particle assembly	GO:0034377	0.00436	APOC1; ABCA7; APOA2; APOE
Regulation of endocytosis	GO:0030100	0.00453	PICALM; APOC1; FCER1G; ABCA7; PLCG2; APOA2; BIN1; APOE; CLU
Sterol transport	GO:0015918	0.00519	APOC1; ABCA7; APOA2; APOE; CLU
Organic hydroxy compound transport	GO:0015850	0.00519	APOC1; ABCA7; APOA2; APOE; CLU
Cholesterol transport	GO:0030301	0.00519	APOC1; ABCA7; APOA2; APOE; CLU
Protein-lipid complex organization	GO:0071825	0.00519	APOC1; ABCA7; APOA2; BIN1; APOE
Regulation of lipid metabolic process	GO:0019216	0.00841	LACTB; APOC1; ABCA7; PLCG2; APOA2; EPHX2; APOE
Negative regulation of amide metabolic process	GO:0034249	0.01440	ABCA7; BIN1; APOE; CELF1; CLU
Negative regulation of amyloid precursor protein catabolic process	GO:1902992	0.01440	PICALM; ABCA7; BIN1; APOE; CLU
Monoatomic cation homeostasis	GO:0055080	0.01440	PICALM; TMEM106B; SLC24A4; TSPOAP1; APOE
Intracellular monoatomic cation homeostasis	GO:0030003	0.01440	PICALM; TMEM106B; SLC24A4; TSPOAP1; APOE

Table 5: Most significant biological processes for MCInc patients obtained from the analysis of the most relevant SNPs with positive IG attributions. In this Table are shown the biological processes, their raw p-value, and the overlap genes.

Biological processes	GO term	p-value	Overlap genes
T cell activation	GO:0042110	0.00695	FCER1G; TREML2; SPI1; RELB
Lymphocyte activation involved in immune response	GO:0002285	0.00695	FCER1G; PLCG2; ERCC1; RELB
Negative regulation of hydrolase activity	GO:0051346	0.00695	PICALM; APOA2; BIN1; CR1
T cell differentiation	GO:0030217	0.00836	FCER1G; SPI1; RELB
Negative regulation of peptidase activity	GO:0010466	0.00836	PICALM; BIN1; CR1
Negative regulation of endopeptidase activity	GO:0010951	0.00836	PICALM; BIN1; CR1
Adaptive immune response based on somatic recombination of immune receptors built from immunoglobulin superfamily domains	GO:0002460	0.01170	FCER1G; CR1L; HLA-DRB1; ERCC1; CR1; RELB
Immune effector process	GO:0002252	0.01800	FCER1G; CR1L; PLCG2; ACE; ERCC1; CR1; RELB
Leukocyte mediated immunity	GO:0002443	0.02020	FCER1G; CR1L; ACE; ERCC1; CR1
Male gonad development	GO:0008584	0.02850	ACE; ERCC1; PLEKHA1
Male sex differentiation	GO:0046661	0.02850	ACE; ERCC1; PLEKHA1
Development of primary male sexual characteristics	GO:0046546	0.02850	ACE; ERCC1; PLEKHA1
Antigen processing and presentation of peptide antigen	GO:0048002	0.02850	FCER1G; ACE; HLA-DRB1
Lymphocyte activation	GO:0046649	0.03080	FCER1G; TREML2; PLCG2; ERCC1; SPI1; RELB
Positive regulation of lymphocyte activation	GO:0051251	0.03400	BLOC1S3; HLA-DRB1; SMARCD3; CR1; SPI1

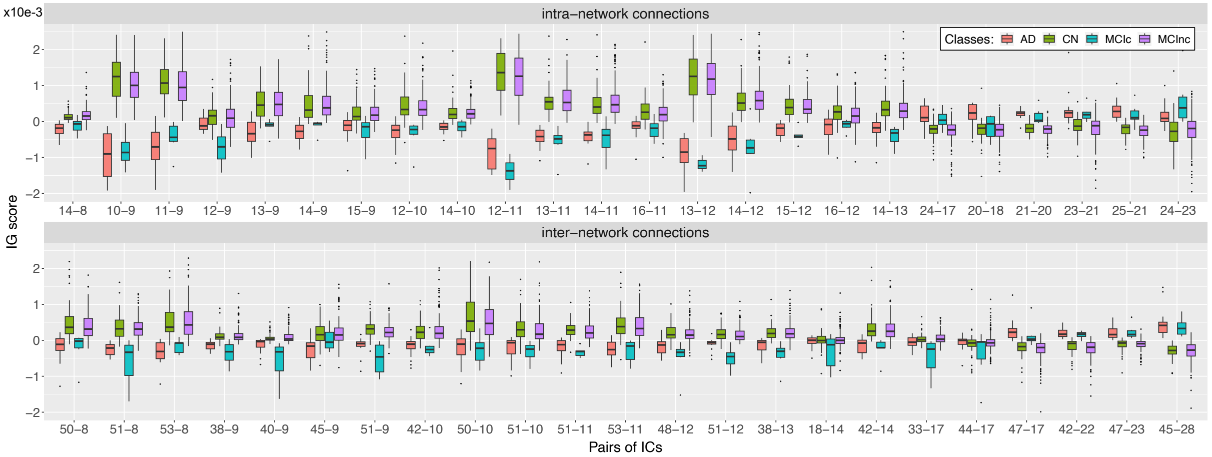

Figure 1: Boxplots that show the distributions of the subjects CN, AD, MCInc, and MCIc in the most important connections based on the fMRI IG score.
