# Supplementary material for: An interpretable generative multimodal neuroimaging-genomics framework for decoding Alzheimer’s disease
Source: ArXiv. 2024 Jun 19:arXiv:2406.13292v1. Preprint. [Version 1] (PMC11213156)
Supplement: Supplementary file 1 [file NIHPP2406.13292v1-supplement-1.pdf]

Supplementary Materials: An interpretable generative multimodal neuroimaging-genomics framework for decoding Alzheimer's disease

Giorgio Dolci[a,b,], Federica Cruciani[a*], Md Abdur Rahaman[b], Anees Abrol[b], Jiayu Chen[b], Zening Fu[b], Ilaria Boscolo Galazzo[a], Gloria Menegaz[a,**], Vince D. Calhoun[b,**], for the Alzheimer's Disease Neuroimaging Initiative[1]

[a] *Department of Engineering and Innovation Medicine, University of Verona, Verona, Italy*
[b] *Tri-Institutional Center for Translational Research in Neuroimaging and Data Science (TReNDS), Georgia State University, Georgia Institute of Technology, Emory University, Atlanta, GA, USA*

arXiv:2406.13292v1 [q-bio.QM] 19 Jun 2024

Preprocessing quality control

A thorough quality control (QC) was performed to retain scans with good normalization to the standard MNI space, which involved discarding sMRI and fMRI images that exhibited low correlation with individual and/or group-level masks. In this spatial correlation process, we first calculated subject-level masks using the subject MRI scans (only the first volume in case of fMRI) by setting the brain voxels to 1 if the values of these voxels were greater than 80% of the average value across whole-brain voxels, and 0 otherwise. Next, after computing the subject-level masks, we calculated a group mask by setting the voxels to 1 for which at least 70% of the subject-level masks had a value of 1. Lastly, we examined the spatial correlations of the subject and group level masks and retained subjects that showed a correlation value greater than 0.85. Additionally, for fMRI, scans with larger head motion parameters ($> 3°$ rotations and > 3 mm translations) were discarded.

*Corresponding author: Department of Engineering and Innovation Medicine, University of Verona, Verona, Italy. *e-mail:* federica.cruciani@univr.it

**Equally contributed as last authors to this work.

[1] Data used in preparation of this article were obtained from the Alzheimer's Disease Neuroimaging Initiative (ADNI) database (adni.loni.usc.edu). As such, the investigators within the ADNI contributed to the design and implementation of ADNI and/or provided data but did not participate in analysis or writing of this report. A complete listing of ADNI investigators can be found at: `http://adni.loni.usc.edu/wp-content/uploads/how_to_apply/ADNI_Acknowledgement_List.pdf`

Results

Table 1: In this Table are reported the correspondences acronym - full name of the sMRI brain regions under analysis.

Acronym	Full name	Acronym	Full name
Ins	Insular Cortex	OFG	Occipital Fusiform Gyrus
TP	Temporal Pole	COpC	Central Opercular Cortex
ScC	Subcallosal Cortex	POpC	Parietal Operculum Cortex
CGp	Cingulate Gyrus, posterior division	Thl	Thalamus
PcC	Precuneous Cortex	Cau	Caudate
FOC	Frontal Orbital Cortex	Put	Putamen
PhGa	Parahippocampal Gyrus, anterior division	Pall	Pallidum
PaGp	Parahippocampal Gyrus, posterior division	Hipp	Hippocampus
LG	Lingual Gyrus	Amy	Amygdala
TFCp	Temporal Fusiform Cortex, posterior division	Acc	Accumbens
TOF	Temporal Occipital Fusiform Cortex		

Table 2: In this Table are reported the 53 ICs present in the sFNC matrices with corresponding brain region name, network at which it belongs to, and spatial location in the brain along X, Y, and Z axis. SC=Sub-cortical; AU=Auditory; SM=sensorimotor; VI=visual; CC=cognitive-control; DM=default-mode; and CB=cerebellar. In *italic* are highlighted the brain regions present in the fMRI connectograms of Results section.

IC ID	Brain region	Network	X	Y	Z	IC ID	Brain region	Network	X	Y	Z
1	Caudate	SC	6.5	10.5	5.5	26	Inferior parietal lobule	CC	45.5	-61.5	43.5
2	Subthalamus/hypothalamys	SC	-2.5	-13.5	-1.5	27	Insula	CC	-30.5	22.5	-3.5
3	Putamen	SC	-26.5	1.5	-0.5	28	*Superior medial frontal gyrus*	CC	-0.5	50.5	29.5
4	Caudate	SC	21.5	10.5	-3.5	29	Inferior frontal gyrus	CC	-48.5	34.5	-0.5
5	Thalamus	SC	-12.5	-18.5	11.5	30	Right inferior frontal gyrus	CC	53.5	22.5	13.5
6	Superior temporal gyrus	AU	62.5	-22.5	7.5	31	Middle frontal gyrus	CC	-41.5	19.5	26.5
7	Middle temporal gyrus	AU	-42.5	-6.5	10.5	32	Inferior parietal lobule	CC	-53.5	-49.5	43.5
8	*Postcentral gyrus*	SM	56.5	-4.5	28.5	33	*Left inferior parietal lobule*	CC	44.5	-34.5	46.5
9	*Left postcentral gyrus*	SM	-38.5	-22.5	56.5	34	Supplementary motor area	CC	-6.5	13.5	64.5
10	*Paracentral lobule*	SM	0.5	-22.5	65.5	35	Superior frontal gyrus	CC	-24.5	26.5	49.5
11	*Right postcentral gyrus*	SM	38.5	-19.5	55.5	36	Middle frontal gyrus	CC	30.5	41.5	28.5
12	*Superior parietal lobule*	SM	-18.5	-43.5	65.5	37	Hippocampus	CC	23.5	-9.5	-16.5
13	*Paracentral lobule*	SM	-18.5	-9.5	56.5	38	*Left inferior parietal lobule*	CC	45.5	-61.5	43.5
14	*Precentral gyrus*	SM	-42.5	-7.5	46.5	39	Middle cingulate cortex	CC	-15.5	20.5	37.5
15	*Superior parietal lobule*	SM	20.5	-63.5	58.5	40	Inferior frontal gyrus	CC	39.5	44.5	-0.5
16	*Postcentral gyrus*	SM	-47.5	-27.5	43.5	41	Middle frontal gyrus	CC	-26.5	47.5	5.5
17	*Calcarine gyrus*	VI	-12.5	-66.5	8.5	42	*Hippocampus*	CC	-24.5	-36.5	1.5
18	*Middle occipital gyrus*	VI	-23.5	-93.5	-0.5	43	Precuneus	DM	-8.5	-66.5	35.5
19	Middle temporal gyrus	VI	48.5	-60.5	10.5	44	*Precuneus*	DM	-12.5	-54.5	14.5
20	*Cuneus*	VI	15.5	-91.5	22.5	45	*Anterior cingulate cortex*	DM	-2.5	35.5	2.5
21	*Right middle occipital gyrus*	VI	38.5	-73.5	6.5	46	Posterior cingulate cortex	DM	-5.5	-28.5	26.5
22	*Fusiform gyrus*	VI	29.5	-42.5	-12.5	47	*Anterior cingulate cortex*	DM	-9.5	46.5	-10.5
23	*Inferior occipital gyrus*	VI	-36.5	-76.5	-4,5	48	*Precuneus*	DM	-0.5	-48.5	49.5
24	*Lingual gyrus*	VI	-8.5	-81.5	-4.5	49	Posterior cingulate cortex	DM	-2.5	54.5	31.5
25	*Middle temporal gyrus*	VI	-44.5	-57.5	-7.5	50	*Cerebellum*	CB	-30.5	-54.5	-42.5
						51	*Cerebellum*	CB	-32.5	-79.5	-37.5
						52	Cerebellum	CB	20.5	-48.5	-40.5
						53	*Cerebellum*	CB	30.5	-63.5	-40.5

Table 3: Most significant biological processes for AD patients obtained from the analysis of the most relevant SNPs with positive IG attributions. In this Table are shown the biological processes, their raw p-value, Benjamini adj. p-value, and the overlap genes.

Biological processes	GO term	p-value	Adj. p-value	Overlap genes
Membrane organization	GO:0061024	$2.124452e^{-05}$	0.02080757	APOA2, CR1, PICALM, MTSS2, PLCG2, RABEP1, NSF, RAB12, NECTIN2, APOE, TOMM40
Regulation of vesicle-mediated transport	GO:0060627	$2.550375e^{-05}$	0.02080757	FCER1G, APOE2, PICALM, SORL1, PLCG2, CDH13, NSF, RAB12, APOE
Regulation of metalloendopeptidase activity involved in amyloid precursor protein catabolic process	GO:1902962	$3.456407e^{-05}$	0.02080757	PICALM, SORL1
Negative regulation of metalloendopeptidase activity involved in amyloid precursor protein catabolic process	GO:1902963	$3.456407e^{-05}$	0.02080757	SORL1, PICALM
Positive regulation of amyloid fibril formation	GO:1905908	0.0001032973	0.04974800	USP8, APOE
Response to lipoprotein particle	GO:0055094	0.0001265865	0.05080336	FCER1G, CDH13, APOE
Cellular response to lipoprotein particle stimulus	GO:0071402	0.0001788776	0.06153390	FCER1G, CDH13, APOE
Negative regulation of amyloid precursor protein catabolic process	GO:1902992	0.000209563	0.06307846	PICALM, SORL1, APOE
Developmental maturation	GO:0021700	0.0002708868	0.06856478	OOSP2, PICALM, SLC24A4, ALDH1A2, BLOC1S3, ERCC2
Positive regulation of complement activation	GO:0045917	0.0003417085	0.06856478	CR1, PBH1
Regulation of aspartic-type endopeptidase activity involved in amyloid precursor protein catabolic process	GO:1902959	0.0003417085	0.06856478	PICALM, SORL1
Negative regulation of metalloendopeptidase activity	GO:1904684	0.0003417085	0.06856478	PICALM, SORL1
Platelet activation	GO:0030168	0.0003701587	0.06856478	FCER1G, PLCG2, APOE, BLOC1S3
Regulation of amyloid-beta formation	GO:1902003	0.0004140288	0.07121295	PICALM, SORL1, APOE
Positive regulation of CoA-transferase activity	GO:1905920	0.000510613	0.07584989	APOA2, APOE

Table 4: Most significant biological processes for MCIc patients obtained from the analysis of the most relevant SNPs with positive IG attributions. In this Table are shown the biological processes, their raw p-value, Benjamini adj. p-value, and the overlap genes.

Biological processes	GO term	p-value	Adj. p-value	Overlap genes
Phospholipid efflux	GO:0033700	$5.528001e^{-07}$	0.001238272	APOA2, ABCA7, APOE, APOC1
Plasma lipoprotein particle assembly	GO:0034377	$4.633589e^{-06}$	0.003459747	APOA2, ABCA7, APOE, APOC1
Protein-lipid complex assembly	GO:0065005	$4.633589e^{-06}$	0.003459747	APOA2, ABCA7, APOE, APOC1
High-density lipoprotein particle assembly	GO:0034380	$2.103289e^{-05}$	0.011778420	APOA2, ABCA7, APOE
Protein-lipid complex subunit organization	GO:0071825	$4.876437e^{-05}$	0.017078497	APOA2, ABCA7, APOE, APOC1
Plasma lipoprotein particle organization	GO:0010873	$4.876437e^{-05}$	0.017078497	APOA2, ABCA7, APOE, APOC1
Susceptibility to T cell mediated cytotoxicity	GO:0060370	$5.380257e^{-05}$	0.017078497	PVR, NECTIN2
High-density lipoprotein particle remodeling	GO:0034375	$6.099463e^{-05}$	0.017078497	APOA2, APOE, APOC1
Amyloid-beta formation	GO:0034205	$8.376335e^{-05}$	0.020847767	PICALM, APH1B, ABCA7, APOE
Positive regulation of phagocytosis	GO:0050766	$9.47894e^{-05}$	0.021232826	FCER1G, APOA2, PLCG2, ABCA7
Cholesterol efflux	GO:0033344	0.0001341986	0.023283667	APOA2, ABCA7, APOE, APOC1
Amyloid-beta metabolic process	GO:0050435	0.0001341986	0.023283667	PICALM, APH1B, ABCA7, APOE
Regulation of sterol transport	GO:0032371	0.0001496329	0.023283667	APOA2, ABCA7, APOE, APOC1
Regulation of cholesterol transport	GO:0032374	0.0001496329	0.023283667	APOA2, ABCA7, APOE, APOC1
Susceptibility to natural killer cell mediated cytotoxicity	GO:0042271	0.0001606363	0.023283667	PVR, NECTIN2

Table 5: Most significant biological processes for MCInc patients obtained from the analysis of the most relevant SNPs with positive IG attributions. In this Table are shown the biological processes, their raw p-value, Benjamini adj. p-value, and the overlap genes.

Biological processes	GO term	p-value	Adj. p-value	Overlap genes
Lymphocyte activation involved in immune response	GO:0002285	$2.662484e^{-05}$	0.02785800	FCER1G, CR1, SPI1, PLCG2, RELB, ERCC1
Mature B cell differentiation involved in immune response	GO:0002313	$4.541461e^{-05}$	0.02785800	CR1, SPI1, PLCG2
Mature B cell differentiation	GO:0002335	$7.16821e^{-05}$	0.02785800	CR1, SPI1, PLCG2
B cell activation involved in immune response	GO:0002312	$7.561089e^{-05}$	0.02785800	CR1, SPI1, PLCG2, ERCC1
Immune effector process	GO:0002252	$7.742635e^{-05}$	0.02785800	FCER1G, APOA2, CR1, CR1L, SPI1, PLCG2, ACE, RELB, ERCC1
Leukocyte mediated immunity	GO:0002443	0.0001351098	0.04050933	FCER1G, CR1, CR1L, SPI1, PLCG2, ACE, ERCC1
Follicular B cell differentiation	GO:0002316	0.0001830395	0.04050933	SPI1, PLCG2
Leukocyte activation involved in immune response	GO:0002366	0.0001887178	0.04050933	FCER1G, CR1, SPI1, PLCG2, RELB, ERCC1
Cell activation involved in immune response	GO:0002263	0.0002026592	0.04050933	FCER1G, CR1, SPI1, PLCG2, RELB, ERCC1
Regulation of complement-dependent cytotoxicity	GO:1903659	0.0004543273	0.07573962	CR1, CR1L
Immunoglobulin mediated immune response	GO:0016064	0.0005441211	0.07573962	FCER1G, CR1, CR1L, ERCC1
B cell mediated immunity	GO:0019724	0.0005752373	0.07573962	FCER1G, CR1, CR1L, ERCC1
Protein transmembrane transport	GO:0071806	0.0006028904	0.07573962	TOMM40L, BLOC1S3, RTN2
Regulation of complement activation, classical pathway	GO:0030450	0.0006337818	0.07573962	CR1, CR1L
Negative regulation of complement activation, classical pathway	GO:0045959	0.0006337818	0.07573962	CR1, CR1L

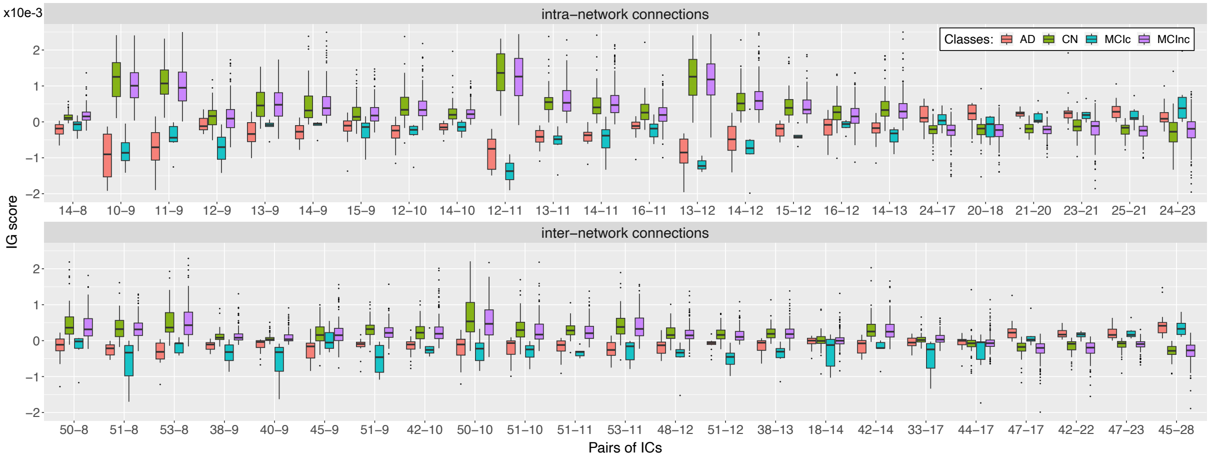

Figure 1: Boxplots that show the distributions of the subjects CN, AD, MCInc, and MCIc in the most important connections based on the fMRI IG score.
